# Associations between personal recovery and service user-rated versus clinician-rated clinical recovery, a cross-sectional study

**DOI:** 10.1186/s12888-022-03691-y

**Published:** 2022-01-18

**Authors:** Regina Skar-Fröding, Hanne Clausen, Jūratė Šaltytė Benth, Torleif Ruud, Mike Slade, Kristin S. Heiervang

**Affiliations:** 1 R&D Department, Division of Mental Health Services, Akershus University Hospital, P.O. box 1000, 1478 Lørenskog, Norway; 2 Norwegian National Advisory Unit on Concurrent Substance Abuse and Mental Health Disorders and Mental Health Division, Innlandet Hospital Trust, Brumunddal, Norway; 3 Institute of Clinical Medicine, Campus Ahus, University of Oslo, Oslo, Norway; 4 Health Services Research Unit, Akershus University Hospital, Lørenskog, Norway; 5 School of Health Sciences, Institute of Mental Health, University of Nottingham, Nottingham, UK; 6 Centre for Medical Ethics, Faculty of Medicine, University of Oslo, Oslo, Norway

**Keywords:** Psychosis, Personal recovery, Clinical recovery, PROM

## Abstract

**Background:**

This study examined the relationship between service user-rated personal recovery and clinician-rated and service user-rated clinical recovery. The relationships between different subdomains of clinical recovery and personal recovery were also assessed.

**Methods:**

In total, 318 mental health service users with a psychosis diagnosis and their clinicians from 39 sites across Norway completed standardized questionnaires regarding personal recovery, clinical symptoms and psychosocial functioning. Regression models were used to investigate the relationship between personal and clinical recovery.

**Results:**

Overall, clinical recovery was positively associated with personal recovery, when rated both by service users and by clinicians. Personal recovery was associated with lower levels of depression, self-harm and problems with relationships when rated by the service users. Among the subdomains rated by the clinicians, personal recovery was associated with fewer problems with relationships and higher aggressiveness.

**Conclusions:**

These findings suggest that affective symptoms are associated with personal recovery, indicating the need for greater focus on depression treatment among people with psychosis. Improving social connections is of importance for personal recovery, and might be an area where clinicians and service users can meet and find agreement on important treatment goals.

## Introduction

The recovery concept originates from two different traditions: the user movement and clinical practice [1]. This duality has resulted in two opposing definitions, known as personal recovery and clinical recovery [2]. Clinical recovery is the definition that has traditionally been the main focus of mental health services, with a focus on symptom reduction and increased functioning [3]. The personal recovery concept as defined by service users differs from this medical conceptualization, and refers to changes in ones attitude to life and the illness with emphasis on hope and the establishment of a meaningful life [3–5]. Connectedness, Hope and optimism, Identity, Meaning and Empowerment (given the acronym CHIME) have been identified as key processes supporting personal recovery [6].

Over the past decade, there has been a growing body of research on the relationship between personal and clinical recovery, with inconsistent findings. Several studies have found either a desynchronized [7], or no relationship [8] between symptom severity and self-reported personal recovery. One study found that although there were no significant correlations between personal recovery and symptom assessments, personal recovery seemed to serve as a protetcting factor by moderating the relationship between positive symptoms and social functioning [9]. Other studies have shown significant correlations between symptom severity and personal recovery [10, 11], and have suggested that even though clinical and personal recovery is not the same, the different concepts of recovery are complementary to each other [8, 10]. A recent meta-analysis on the relationship between personal and clinical recovery found a small to-medium association between overall symptom severity and personal recovery [12]. Insight into this association is important because it may inform mental health services what treatment strategies to provide.

As empirical research on the two concepts is growing, the complexity of the relationship between them has become more evident. For example, among different subdomains of clinical recovery, affective symptoms have been shown to play a significant role in relation to personal recovery [10, 13] and subjective quality of life [14, 15], a concept closely related to personal recovery. In the meta-analysis of personal and clinical recovery, affective symptoms were shown to play a more important role for personal recovery than positive or negative symptoms [12]. More research is needed to gain a better understanding of whether the attainment of some elements of recovery is dependent on the attainment of others, and if so, to identify important factors that affect the process of personal recovery. This will have implications for the future development of recovery-oriented practices.

However, research on the relationship between personal and clinical recovery often reflects this dichotomized view of recovery, with clinicians rating clinical recovery aspects and service users reporting their personal recovery. It has recently been argued that it might be meaningful to assess both service user and staff perspectives on clinical recovery alongside service user-rated assessments of personal recovery in mental health research [7]. Investigating if and how self-reported clinical domains are associated with personal recovery could reveal important aspects for personal recovery. Service users and clinicians have independent perspectives on clinical recovery, and service users have differing perspectives on clinical and personal recovery [7, 16], a complexity that needs to be reflected in health service research design. Most research that has aimed to disentangle the complex relationship between personal and clinical recovery has only included clinician- or researcher-assessed clinical symptoms [10, 12, 17]. Examining the role of service user-rated clinical symptoms in relation to personal and clinical recovery is relevant and could shed light on the relationship between these two concepts, thereby revealing new areas of clinical importance.

The aim of this study was to examine the relationship between personal recovery reported by service users and clinical recovery rated by both clinicians and service users. To that end, we addressed the following research questions: Is there a relationship between personal recovery and clinical recovery as rated by clinicians? Is there a relationship between personal recovery and clinical recovery as rated by service users? Is there a relationship between personal recovery and different subdomains of clinical recovery when rated by clinicians and service users?

## Methods

### Design

This cross-sectional study used baseline data from the Norwegian research project *A pairwise randomized study on implementation of guidelines and evidence-based treatments of psychoses* (ClinicalTrials NCT03271242). This project is a cluster randomized trial focusing on the current implementation of the Norwegian national clinical guidelines for the treatment of psychosis, and on how the implementation of evidence-based treatments can be improved. The study was approved by the Regional Committee for Medical and Health Research Ethics (REK Sørøst B 2015/2169), and followed the principles laid down in the Declaration of Helsinki.

### Setting and sample

A total of 325 mental health service users from six health authorities across Norway, including three university hospitals, were recruited. Thirty-nine clinical units and hospital departments with outpatient clinics, day units, mobile teams, and inpatient wards participated. The inclusion criteria were mental health service user aged 16 years or older and diagnosed with psychosis (ICD-10 F20–29) [18]. The exclusion criterion was the inability to read or understand Norwegian. Service users with missing data (*n* = 7) were excluded, reducing the final study sample to *N* = 318.

The large majority of the participating clinical units were local community mental health centres (CMHCs). The CMHCs consists of outpatient clinics, mobile teams and inpatient wards, and have multidisciplinary clinical staff. The clinical staff in the CMHCs includes psychiatrists, clinical psychologists, mental health nurses and several other professional groups [19]. The participating clinicians in this study were the ones with the closes relationships to the service users, and who were responsible for providing treatment and case management.

### Measures

#### Service user-rated personal recovery

The outcome measure used in this study to examine personal recovery was the *Questionnaire about the Process of Recovery (QPR)* [20], a 15-item self-report measure of recovery with adequate psychometric properties developed in collaboration between clinicians and service user researchers [21]. QPR was included as a measure of level of personal recovery because it is one of the most wellknown and widely used scales for measuring personal recovery. It is also one of the personal recovery measures with strongest evidence base, and the one that most closely maps to the CHIME framework of recovery [22]. Items are rated on a 5-point Likert scale (0, “disagree strongly”; 1, “disagree”; 2, “neither agree nor disagree”; 3, “agree”; 4, “agree strongly”). The total sum score ranges from 0 (low recovery) to 60 (high recovery). Psychometric evaluation of the QPR in the current sample showed a one-factor solution with high scale reliability (Cronbach’s alpha 0.91).

#### Service user-rated clinical recovery

*The Behavior and Symptom Identification Scale (BASIS-24)* is a brief service user self-report measure of psychopathology and functioning that was developed to assess mental health treatment outcomes. It consists of 24 items assessing the following six symptoms and functioning domains: “depression/functioning”, “interpersonal relationships”, “self-harm”, “emotional lability”, “psychosis”, and “substance abuse”. The scale has shown good validity and reliability for assessing mental health status and functioning from the perspective of service users [23, 24]. The BASIS-24 was chosen as a measure of clinical recovery, because it is one of the most frequently used patient-reported instruments to evaluate mental health and psychosocial functioning [25], with good validation of symptoms of psychosis [23]. The six domains were included as clinical recovery subdomains and the sum scores of all six domains were included as main measures of service user-rated clinical symptoms. Scores were calculated as described in the BASIS-24 instruction guide [26], providing a score between 0 and 4 with higher scores indicating more severe problems.

#### Clinician-rated clinical recovery

The Health of the Nation Outcome Scale (HoNOS) [27] is a 12-item staff-rated measure of mental health and psychosocial functioning. Each item is rated on a 5-point severity scale from 0 (no problem) to 4 (severe to very severe problem). The scale was developed to measure outcomes routinely for adults with mental illness. It is a widely used routine outcome measure in mental health services in many countries [28], and has been regarded as adequate for assessing outcomes for different service user groups on a range of mental health-related constructs, and for routinely monitoring outcomes [29]. The total score (0–48) of all 12 items was included as the main measure of clinician-rated clinical recovery, while nine of the 12 items were included as clinical recovery subdomains variables. The three items not included (physical illness or disability problems, problems with living conditions and problems with occupation and activities) were excluded because they were considered to measure somatic health and actual access to resources rather than clinical recovery. The clinicians were instructed to complete a net-based training course of the HoNOS scale and the instruction manual was included in the questionnaire.

*The Global Assessment of Functioning Scale (GAF)* is a standardized measure assessing impairments caused by mental factors [30]. Clinicians rate the level of functioning and severity of service users’ symptoms on a scale between 1 and 100 with lower scores indicating more severe symptoms and a lower level of functioning. The split version of the scale used in this study has two subscales: symptom (GAF-S) and function (GAF-F) [31]. It was mandatory to use the GAF scales in the participating mental health clinics at the time of the study, and each clinic were responsible for training their clinicians.

### Covariates

Age and gender were included as covariates in the analyses.

### Procedure

Service users were recruited by clinicians working at the participating mental health units. Eligible service users already in contact with the clinic at the time, and newly referred service users assessed to have psychosis, were asked to participate. Only participants who gave written informed consent were included. All participants were evaluated to be capable of giving informed consent.

The assessments of the service users were provided by the clinicians and teams who were responsible for providing treatment and case management, and who had the closest relationships with the service users. The questionnaires were administered to the service users by the clinicians or other personnel in the teams at the clinics.. Service users were given a place to sit and fill out the questionnaire or took it home with them. When finished, the questionnaires were placed in a sealed envelope, and returned to the clinic. The recruitment period lasted from June 2016 to March 2017.

### Analysis

The sociodemographic and clinical service user characteristics are presented as frequencies and percentages or means and standard deviations (SDs), as appropriate (Table 1). Pearson’s correlations among the sum/total and subdomain scores of BASIS-24 and HoNOS were calculated to assess the extent to which these scales correlated (Table 2).

**Table 1:** Sociodemographic characteristics of the participants (*N* = 318)

Characteristics	
Gender N (%)	
Female	130 (41)
Male	187 (59)
Ethnicity N (%)	
Norwegian	274 (88)
Other	39 (12)
Age (years) mean (SD)	40 (12.7)
Diagnosis N (%)	
Schizophrenia	145 (53)
Schizoaffective disorder	54 (20)
Other	74 (27)
Community Treatment Order N (%)	
Yes	42 (13)
No	269 (87)
GAF symptom mean^a^ (SD)	52 (13.0)
GAF function mean^a^ (SD)	51 (11.4)
QPR total score mean^b^ (SD)	41 (10.3)
BASIS-24 total score mean^c^ (SD)	1.21 (0.66)
Emotional lability mean (SD)	1.55 (0.90)
Psychosis mean (SD)	1.05 (1.04)
Depression/functioning mean (SD)	1.31 (0.94)
Relationships mean (SD)	1.60 (0.98)
Self-harm mean (SD)	0.34 (0.65)
Substance abuse mean (SD)	0.43 (0.68)
HoNOS total score mean^d^ (SD)	7.67 (4.83)
Aggressiveness mean (SD)	0.35 (0.61)
Non-accidental self-injury mean (SD)	0.17 (0.51)
Problem drinking or drug-taking mean (SD)	0.36 (0.88)
Cognitive problems mean (SD)	0.85 (0.80)
Hallucinations and delusions mean (SD)	1.08 (1.16)
Depressed mood mean (SD)	0.87 (0.90)
Other mental and behavioural problems mean (SD)	1.33 (1.15)
Problems with relationships mean (SD)	1.58 (1.10)
Problems with activities related to daily living mean (SD)	1.09 (0.97)

^a^ The Global Assessment of Functioning Scale (GAF) split version

^b^ Questionnaire about the Process of Recovery (QPR)

^c^ The Behavior and Symptom Identification Scale (BASIS-24)

^d^ Health of the Nation Outcome Scale (HoNOS)

**Table 2:** Correlations between BASIS-24 and HoNOS total score and the subdomains

Correlations	Basis-24 sum score	Substance abuse	Self-harm	Depression/functioning	Psychosis	Emotional lability	Relationships
HoNOS total score	.52	.26	.31	.49	.40	.28	.22
Aggressiveness	.21	.15	.01	.17	.12	.18	.08
Non-accidental self-injury	.27	−.03	.37	.23	.15	.22	.06
Problem drinking or drug-taking	.13	.60	.04	.05	.16	−.01	.07
Cognitive problems	.20	.05	.19	.19	.22	.09	.10
Hallucinations and delusions	.34	.08	.18	.28	.50	.19	.11
Depressed mood	.40	.08	.26	.40	.21	.24	.18
Other mental and behavioral problems	.41	.11	.24	.43	.28	.25	.10
Problems with relationships	.36	.16	.15	.35	.18	.12	.27
Problems with activities related to daily living	.34	.07	.20	.34	.19	.19	.19

Five linear regression models were estimated to assess the association between personal recovery (QPR) and service user-rated clinical recovery (BASIS-24) and clinician-rated clinical recovery (HoNOS). The BASIS-24 sum and subdomains scores were included in Model 1 and Model 2, respectively (Table 3). The HoNOS total score and the nine pre-chosen HoNOS items were included in Model 3 and Model 4, respectively (Table 4). The sum and subdomains scores of the two measures were analysed in separate models, since including them in the same model would imply multicollinearity issues. Finally, the BASIS-24 sum score and the HoNOS total score were included in Model 5 (Table 5). GAF-symptom, GAF-function, age and gender were entered as covariates in all the models. Bivariate and multiple models were estimated. The intra-class correlation coefficient was estimated to assess the degree of clustering due to data collection from different mental health units. As no cluster effect was identified, no adjustment was needed in the regression models.

**Table 3:** Results of linear regression models for the association between personal recovery (QPR) and service user-rated clinical recovery (BASIS-24)

Variables	Model 1, *N* = 295						Model 2, *N* = 305					
	Bivariate models			Multiple model			Bivariate models			Multiple model		
	Regr. coeff	95% CI	p	Regr.coeff	95% CI	p	Regr. coeff	95% CI	p	Regr. coeff	95% CI	p
BASIS-24 sum score	−8.51	−10.31;-6.99	**.001**	−8.47	− 10.12;-6.83	**.001**						
Emotional lability							−3.01	−4.26;-1.75	**.001**	0.67	− 0.67;2.01	.325
Psychosis							−2.85	−3.91;-1.78	**.001**	−0.12	−1.21;0.97	.829
Depression/functioning							−5.36	−6.46;-4.27	**.001**	−3.89	− 5.31;-2.47	**.001**
Relationships							−4.34	−5.41;-3.27	**.001**	−3.00	−4.06;-1.95	**.001**
Self-harm							−5.61	−7.41;-3.82	**.001**	−2.61	−4.41;-0.82	**.004**
Substance abuse							−1.70	−3.35;-0.05	.**044**	− 0.53	−2.01;0.95	.481
Age	0.02	−0.07;;3.09	.558	−0.01	−2.05;2.03	.993	0.04	−0.06;0.13	.444	−0.02	−0.10;0.06	.577
GAF-symptom	0.11	0.02;0.20	.**015**	−0.02	− 0.13;0.08	.662	0.10	0.01;0.19	**.024**	−0.01	−0.11;0.09	.803
GAF-function	0.19	0.09;0.29	.**001**	0.05	−0.07;0.17	.446	0.19	0.09;0.29	**.001**	0.03	−0.09;0.15	.620
Gender												
Male-ref	0						0			0		
Female	0.71	−1.67;3.09	.558	−0.01	−2.05;2.03	.993	0.81	−1.53;3.15	.497	−0.74	−2.80;1.32	.482

**Table 4:** Results of linear regression model for associations between personal recovery (QPR) and clinician–rated clinical recovery (HoNOS)

Variables	Model 3, *N* = 246						Model 4, N = 246					
	Bivariate models			Multiple model			Bivariate models			Multiple model		
	Regr. coeff	95% CI	p	Regr. coeff	95% CI	p	Regr. coeff	95% CI	p	Regr. coeff	95% CI	p
HoNOS total score	−0.66	− 0.91; − 0.40	**.001**	− 0.57	− 0.89; − 0.25	**.001**						
Aggressiveness							−0.19	−2.41; 2.02	.864	2.41	0.08; 4.74	**.043**
Non-accidental self-injury							−3.23	−5.60; −0.86	**.008**	−1.38	−3.90; 1.14	.283
Problem drinking or drugtaking							−0.93	−2.39; 0.54	.216	−0.24	−1.73; 1.25	.750
Cognitive problems							−1.92	−3.48; −0.35	**.017**	−0.39	−2.13; 1.36	.665
Hallucinations and delusions							−1.79	−2.88; −0.69	**.002**	−0.83	−2.10; 0.43	.195
Depressed mood							−2.60	−3.98; −1.22	**.001**	−0.82	−2.52; 0.87	.340
Other mental and behavioral problems							−1.63	−2.73; −0.52	**.004**	0.13	−1.34; 1.59	.865
Problems with relationships							−3.11	−4.21; −2.00	**.001**	−2.28	−3.69; −0.87	**.002**
problems with activities related to daily living							−2.16	−3.46; − 087	**.001**	−0.19	−1.81; 1.43	.818
Age	0.04	−0.06; 0.13	.479	−0.01	−0.11;0.09	.858	0.04	−0.06; 0.13	.479	0.03	- 0.08; 0.13	.611
GAF-symptom	0.13	0.03; 0.23	**.009**	−0.06	−0.21; 0.09	.425	0.13	0.03; 0.23	**.009**	−0.05	−0.21; 0.11	.508
GAF-function	0.20	0.10; 0.31	**.001**	0.12	−0.05; 0.29	.174	0.20	0.10; 0.31	**.001**	0.11	−0.08; 0.29	.259
Gender												
Male-ref	0			0				0			0	
Female	1.64	−0.93; 4.20	.209	0.90	−1.61; 3.41	.482	1.64	−0.93; 4.20	.209	0.76	−1.82; 3.34	.562

**Table 5:** Results of linear regression model for associations between personal recovery (QPR), clinician–rated clinical recovery (HoNOS) and service user-rated clinical recovery (BASIS-24)

Variables	Model 5, *N* = 235					
	Bivariate models			Multiple model		
	Regr. coeff	95% CI	P	Regr. coeff	95% CI	p
BASIS-24 sum score	−8.16	−9.90; −6.43	**.001**	−7.87	−9.97; −5.77	**.001**
HoNOS total score	−0.64	−0.91; −0.38	.**001**	−0.07	− 0.39;0.26	.688
Age	0.01	−0.09; 0.12	.795	−0.04	−0.13;0.05	.386
GAF-symptom	0.15	0.05; 0.25	**.005**	−0.03	−0.17; 0.12	.714
GAF-function	0.21	0.10; 0.32	**.001**	0.04	−0.12; 0.20	.635
Gender						
Male-ref	0			0		
Female	1.43	−1.23; 4.08	.291	0.28	−2.07; 2.64	.813

All tests were two-sided, and results with *p*-values ≤0.05 were considered statistically significant.

Imputation of missing values on the GAF-S (*n* = 39), GAF-N (n = 39) and QPR (*n* = 24) was performed by first generating the empirical distribution for existing values. A random number was drawn from that distribution and used to replace the missing value. The process was repeated until all missing values were imputed. All regression models were estimated for service users with no missing values on the included covariates.

Due to many missing values, mainly in the HoNOS scale (*N* = 65), those included and not included in the regression analyses were compared. The differences between continuous variables (QPR, BASIS-24 sum score, GAF-S, GAF-F and age) were assessed by independent sample t-tests, while categorical variables (gender, diagnosis, ethnicity and being under a Community Treatment Order) were compared by χ2-tests.

## Results

### Sample characteristics

The mean age of the 318 participants was 40 years (SD = 12.7) and 41% (*n* = 130) were female. The majority of the participants were Norwegian (*n* = 274, 88%), and 53% (*n* = 145) had a diagnosis of schizophrenia. A more detailed description of the participants’ sociodemographic characteristics is shown in Table 1.

### Correlations between BASIS-24 and HoNOS total scores and subdomains

Pearson’s correlations assessing the association between the BASIS-24 and HoNOS scales are presented in Table 2. The results showed weak to moderately strong correlations (ranging from −.01 to .60).

### Relationship between personal recovery and service user-rated clinical recovery

Table 3 shows the results from the regression analyses assessing the association between personal recovery and service-user rated clinical symptoms. In the multiple model, a higher general level of service user-rated clinical recovery (lower BASIS-24 sum score) was significantly associated with higher personal recovery (higher QPR score) (Model 1). Among the clinical subdomains, lower scores on depression/functioning and self-harm and fewer problems with relationships were significantly associated with higher personal recovery (Model 2).

### Relationship between personal recovery and clinician-rated clinical recovery

Table 4 shows the results from the regression analyses assessing the association between personal recovery and clinician-rated clinical symptoms. In the multiple model, higher clinician-rated clinical recovery (HoNOS total score) was significantly associated with higher personal recovery (Model 3). Among the clinical subdomains, fewer problems with relationships and higher aggressiveness were significantly associated with higher personal recovery (Model 4).

### Relationship between personal recovery and service user-rated vs clinician-rated clinical recovery

In the bivariate analyses (Table 5), personal recovery seemed to be associated with both clinical recovery when reported by service users and clinicians. However, personal recovery was more strongly related with service user-rated clinical recovery than with clinician-rated recovery, as shown in the multiple analysis. This was also supported by standardized regression coefficients (not shown).

No significant differences were found between the dropouts and the remaining participants when compared by independent sample t-tests and χ2-tests.

## Discussion

The present study examined the relationship between personal recovery and clinical recovery and its subdomains, as rated by clinicians and service users. The results revealed that personal recovery was significantly associated with clinical recovery, as rated by both service users and clinicians.

Among the service user-rated clinical subdomains, fewer depressive symptoms and everyday coping (depression/functioning), being able to manage social situations and having other people to turn to (Problems with relationships), and fewer suicidal thoughts/thoughts about self-harm (Self-harm) were related to higher personal recovery. Neither the service user- nor clinician-rated subdomain of psychotic symptoms showed any significant associations with personal recovery. This finding is consistent with previous studies showing that affective symptoms are more strongly associated with personal recovery than are other psychosis-specific symptoms, such as hallucinations and delusions [12]. However, it has been suggested that psychotic symptoms may increase distress, which in turn, has a negative influence on personal recovery [32]. Therefore, the role of depression as a potential mediating variable behind factors related to personal and clinical recovery among service users with psychosis should be further investigated. This finding also has clinical implications, providing further support for the already highlighted need for more investigation and treatment for depressive symptoms among people with psychosis [33]. As depression among people with psychosis has been consistently and robustly linked to insight [34], the role of insight in the relationship between depression and personal recovery should be further investigated.

Among the clinician-rated clinical subdomains, fewer problems with relationships and higher aggressiveness were significantly associated with higher personal recovery. Our study show that, the results differ when clinicians and service users report on the importance of different clinical subdomains for personal recovery. When service users report on this, it seems that the subdomains regarding depression and related themes such as self-harm and suicidal thoughts play an important role in personal recovery; this does not appear when rated by clinicians. This could be due to an underestimation of affective symptoms and the fact that affective symptoms often are seldom given enough consideration in clinical treatment, as highlighted by previous research on the role of depression in schizophrenia [33]. However, problems with relationships appeared significant when reported by both clinicians and service users. This finding supports previous research, which has demonstrated the importance of social contact for personal recovery [6, 35]. It is also clinically relevant, emphasizing the need for health-care services to facilitate the building and maintenance of a strong social supportive system for individuals with psychosis. Improving social connections can both be aimed at the individual level such as strengthening the individuals’ relations to friends and family, and at a more structural level such as being part of the society. For example, reducing conflict and strengthen social support from family members as an intervention for people with psychosis have a strong evidence-base when it comes to clinical recovery [36]. However, the implementation level of structured family interventions for people with psychosis are poor, in Norway [37] as well as internationally [38]. Other interventions with a significant evidence base worth mentioning are Individual Placement and Support approach to employment and the development of Recovery Colleges [39]. Our results show that improving social relationships might be an area of great relevance for personal recovery, as it is supported by findings from both clinicians and service users. The importance of other people, the social environment, and society for personal recovery has been a topic of discussion, with some defining it as a separate kind of recovery, termed “social recovery” [40], while others define it as a part of personal recovery, or even as a part of clinical recovery in terms of functioning. Regardless of how it is conceptualized, improving social connections seems to be an important area for mental health services to focus on to strengthen the personal recovery of service users with psychosis. In addition, social support has proven to be related to both subjective and objective markers of recovery [41].

The finding that clinician-rated higher aggressiveness was significantly associated with higher personal recovery, was surprising and unexpected. To our knowledge, this association has not been reported in any previous studies. Perhaps this finding reflects a high level of assertiveness in the person, which in turn could be associated to an ability for mobilization and a strive for a better life. However, the finding could be a sign of overestimation in the regression model (as pointed out in the limitation section). Another surprising finding is that problems with alcohol or drugs did not appear to be significantly related to personal recovery in our sample, which should be further investigated.

Our findings suggest that personal recovery is more strongly related to service user-rated clinical recovery than to clinician-rated recovery. However, the service-user domains of importance are primarily social relations and depressive symptoms. This is of clinical importance, as it shows that from the user perspective, these two aspects are more important for personal recovery than are typical psychosis-specific symptoms such as hallucinations and delusions.

### Strengths and limitations

A major strength of this study is the broad group of participants with psychosis that were recruited from “real-world” clinical practice in many different units, which increases the generalizability of the results. However, as the study participants were not randomly selected, the sample might not be representative of the Norwegian population of individuals with psychosis. Therefore, our results should be interpreted with caution. One possible limitation of the study is the common rater effect, a known potential bias when including several measures from the same respondent. However, correlation analysis between QPR and BASIS-24 total score showed only a moderate correlation, speaking against such bias. In addition, two different measures were used to assess clinical recovery were used, which might have introduced some uncertainty in our comparisons. In addition, the unexpected significant finding of the “aggressiveness” subscale might be a sign of overestimation in the model; this should be taken into consideration. Unfortunately, there are no detailed demographics about the participating clinicians, which should be considered a limitation of the study. Finally, given the cross-sectional nature of this study, no casual interpretations were possible.

## Conclusions and implications

Clinical recovery was significantly associated with personal recovery when rated by both clinicians and service users, but more strongly when rated by service users. The results differed when clinicians and service users reported on the clinical subdomains. Service user-reported depression and related themes such as self-harm and suicidal thoughts were associated with personal recovery; this association did not appear when rated by clinicians. In addition, neither service user-rated nor clinician-rated psychotic symptoms showed any significant associations with personal recovery. These findings suggest that affective symptoms are more strongly associated with personal recovery than are psychosis-specific symptoms such as hallucinations and delusions. This finding has clinical implications, suggesting the need for greater focus on treatment for depression among people with psychosis. However, problems with relationships appeared significant when reported by both clinicians and service users. This finding indicates that improving social connections might be an area of clinical importance when it comes to strengthening the personal recovery of service users with psychosis, and that it is important for mental health-care services to facilitate the building and maintenance of a strong social support system for individuals with psychosis.

## Data Availability

The datasets used and/or analysed during the current study are available from the corresponding author on reasonable request.
